# Trypsin, Tryptase, and Thrombin Polarize Macrophages towards a Pro-Fibrotic M2a Phenotype

**DOI:** 10.1371/journal.pone.0138748

**Published:** 2015-09-25

**Authors:** Michael J. V. White, Richard H. Gomer

**Affiliations:** Department of Biology, Texas A&M University, College Station, Texas, United States of America; University of Kansas Medical Center, UNITED STATES

## Abstract

For both wound healing and the formation of a fibrotic lesion, circulating monocytes enter the tissue and differentiate into fibroblast-like cells called fibrocytes and pro-fibrotic M2a macrophages, which together with fibroblasts form scar tissue. Monocytes can also differentiate into classically activated M1 macrophages and alternatively activated M2 macrophages. The proteases thrombin, which is activated during blood clotting, and tryptase, which is released by activated mast cells, potentiate fibroblast proliferation and fibrocyte differentiation, but their effect on macrophages is unknown. Here we report that thrombin, tryptase, and the protease trypsin bias human macrophage differentiation towards a pro-fibrotic M2a phenotype expressing high levels of galectin-3 from unpolarized monocytes, or from M1 and M2 macrophages, and that these effects appear to operate through protease-activated receptors. These results suggest that proteases can initiate scar tissue formation by affecting fibroblasts, fibrocytes, and macrophages.

## Introduction

The failure of wounds to heal properly constitutes a major medical problem, with both acute and chronic wounds consuming treatment time and resources [1, 2]. The opposite of poorly healing wounds is fibrosis, where unnecessary and inappropriate scar tissue forms in an organ [3]. Fibrosing diseases include pulmonary fibrosis, congestive heart failure, liver cirrhosis, and end stage kidney disease, and are involved in 45% of deaths in the United States [4]. A key question in wound healing and fibrosis is the triggering mechanism that induces scar tissue formation.

One of the events preceding scar tissue formation in a healing wound is the clotting cascade, in which the protease thrombin cleaves fibrinogen to fibrin. Thrombin activity is upregulated immediately after wounding [5] and in fibrotic lesions [6]. Mast cells are found in both fibrotic lesions and sites of wound healing [7–9]. Mast cells degranulate to release tryptase, and tryptase is upregulated in wounds and fibrotic lung tissue [7–12]. Tryptase and thrombin, as well as other proteases such as trypsin, potentiate wound healing and scar tissue formation by increasing fibroblast proliferation and collagen secretion [9, 13–15], inducing platelet aggregation [16], and by potentiating the differentiation of monocytes into fibroblast-like cells called fibrocytes [17, 18]. Although the term fibrocyte has been used to designate circulating CD34+, CD45+, and collagen-positive cells [19], in this report we adhere to the original definition of fibrocyte as a monocyte-derived, tissue-resident cell [20]. Thrombin signals through protease-activated receptor-1 (PAR-1), and trypsin and tryptase signal through protease-activated receptor-2 (PAR-2) [9, 21–23], and we found that agonists of PAR-1 and PAR-2 potentiate fibrocyte differentiation [18].

In addition to differentiating into fibrocytes, monocytes can differentiate into classically-activated M1 macrophages or alternatively-activated M2 macrophages [24]. M1 macrophages are associated with pathogen responses, and M2 macrophages are associated with immuno-regulation and tissue restructuring [25, 26]. There are at least two subpopulations of M2 macrophages. Mreg macrophages have an anti-inflammatory phenotype, and do not secrete matrix proteins [24]. M2a macrophages are involved in scar tissue formation in both wound healing and fibrosis [27–30]. M2a macrophages become more prevalent as wound healing progresses and collagen deposition increases, and directly secrete the matrix protein fibronectin, a major component of scars [31–33]. Removal of macrophages from a mouse wound by depletion or conditional knockout lowers the amount of scar tissue deposited in the wounds [34], indicating that macrophages play a major role in wound healing [35]. Depletion of macrophages from mice also lowers the amount of scar tissue formed after induced liver fibrosis, indicating that macrophages also participate in the progression of fibrosis [36].

Human M1, M2a, and Mreg macrophages, while morphologically similar, display different surface markers and secrete different cytokines [24]. CD163 is a marker of M2 macrophage differentiation that is sometimes classed as an Mreg marker [25, 37]. Fibronectin is an unambiguous marker of M2a macrophage differentiation [38]. CD206 is sometimes classed as an Mreg marker, and sometimes as an M2a marker [25, 37]. CCR7 is a commonly used marker for M1 macrophages [25]. M1 and M2 macrophages also have different secretion profiles, with M1 macrophages secreting higher levels of the cytokine IL-12 compared to M2 macrophages [24]. M2 regulatory macrophages secrete increased levels of the anti-inflammatory cytokine IL-10 [24]. M2a macrophages secrete intermediate amounts of IL-12 and IL-10, and high amounts of IL-4 and IL-13 [24, 39]. Polarized macrophages display a spectrum of markers, and macrophage phenotypes can only be assessed by examining multiple differentiation markers [24].

In this report, we show that trypsin, tryptase and thrombin bias populations of human monocytes, M1 macrophages, or M2 regulatory macrophages towards an M2a phenotype, suggesting an additional mechanism whereby blood clotting, and/or mast cell degranulation, releases and/or activates extracellular proteases to induce and/or potentiate wound healing and fibrosis.

## Materials and Methods

### Proteases

Tosyl phenylalanyl chloromethyl ketone (TPCK)-treated bovine trypsin (10,000 BAEE units/mg, Sigma, St. Louis, MO) and human thrombin (1000 NIH units/mg, Sigma) were resuspended following the manufacturer’s instructions. Tryptase purified from human mast cells (70 BPVANA units/mg, Fitzgerald, Acton, MA) was mixed with 15 kDa heparin from porcine stomach (Sigma) in a 1:10 molar ratio of tryptase to heparin immediately after thawing [40].

### Immunohistochemistry and ELISAs

Human blood was collected from volunteers who gave written consent and with specific approval from the Texas A&M University human subjects Institutional Review Board. PBMC were isolated and cultured as previously described [41] to differentiate fibrocytes and macrophages in serum-free media (SFM), composed of Fibrolife basal media (Lifeline Cell Technology, Walkersville, MD) supplemented with 10 mM HEPES (Sigma, St. Louis, MO), 1× non-essential amino acids (Sigma), 1 mM sodium pyruvate (Sigma), 2 mM glutamine (Lonza, Basel, Switzerland), 100 U/ml penicillin and 100 μg/ml streptomycin (Lonza), and ITS-3 (Sigma), composed of 10 μg/ml recombinant human insulin, 5 μg/ml recombinant human transferrin, and 550 μg/ml filter-sterilized human albumin. PBMC were isolated and cultured as previously described [42] to polarize macrophages towards M1 and M2 phenotypes, with the following modifications. PBMC were cultured with 25 ng/ml MCSF or GMCSF for one week in 10% serum, as previously described [42], after which the cells were treated with 12.5 ng/ml trypsin, tryptase, or thrombin for two days. Protease-activated receptor-1 agonist SFLLRN-NH2 (American Peptide, Sunnyvale, CA) and protease-activated receptor-2 agonist 2f-LIGRL-NH2 (EMD Millipore) were added to PBMC cultures at 10 μM, as previously described [41]. Protease-activated receptor-1 inhibitor SCH 79797 (Axon Medchem, Reston, VA, added to cells at 5 μg/ml) and protease-activated receptor-2 inhibitor ENMD-1068 (Enzo Life Sciences, Farmingdale, NY; 50 μg/ml) were used to block PAR-1 and PAR-2 signaling, as previously described [41]. Cells were fixed and stained for CCR7 (mouse monoclonal clone 150503, R&D systems, Minneapolis, MN), CD163 (mouse monoclonal clone GH1/61, Biolegend, San Diego, CA), CD206 (mouse monoclonal clone 15–2, Biolegend), and fibronectin (rabbit polyclonal, Sigma), as previously described [43]. Fibrocytes and macrophages were counted based on their morphology, as previously described [44]. For each donor and each stain, at least 200 macrophages and at least 100 fibrocytes were scored. Conditioned media from PBMC cultured with or without 12.5 ng/ml protease were analyzed for IL-10, IL-12, and IL-4 and IL-13 using a human IL-12 ELISA kit (Peprotech, Rocky Hill, NJ), a human IL-10 ELISA kit (Biolegend), a human IL-4 ELISA kit (Peprotech, Rocky Hill, NJ), and a human IL-13 ELISA kit (Peprotech, Rocky Hill, NJ), following the manufacturer’s instructions.

### Staining intensity measurements

Images of cells were obtained with a Nikon D1X SLR camera (Nikon, Tokyo, Japan) or a 10 MP USB camera (OMAX, Kent, WA) imager on a Nikon diaphot inverted tissue culture microscope (Nikon) with a 10x lens. The image analysis program CellProfiler [45] was used to identify cells in the images as either macrophages or fibrocytes (based on their elongated shape), and measure the relative mean staining intensity of each cell. For each donor and each stain, at least 5 fields of view, comprising about ~200 cells, were analyzed. The CellProfiler pipeline is in the supplemental methods section.

### Co-culture of fibroblasts and monocytes

Normal adult dermal fibroblasts (Lonza, 2000 cells per well) and PBMC (50,000 cells per well) were cultured in 96 well plates in 200 μl SFM for 5 days. The media was removed, plates were dried, and were fixed and stained using Masson’s trichrome kit (Sigma, HT15), following the manufacturer’s directions, but omitting the staining with Weigert’s hematoxylin. The plates were air dried, and staining intensity was measured by absorbance at 455 and 700 nm using a Synergy MX plate reader (Biotek, Winooski, VT).

### Statistics

Statistics were performed using Prism (Graphpad Software, San Diego, CA). Differences were assessed by two-tailed unpaired and two-tailed paired t-tests, where indicated. Paired t-tests were used to assess significance in highly variable experiments. Significance was defined by p<0.05.

## Results

### Trypsin, tryptase, and thrombin potentiate the differentiation of monocytes into M2a macrophages

**Fig 1:**
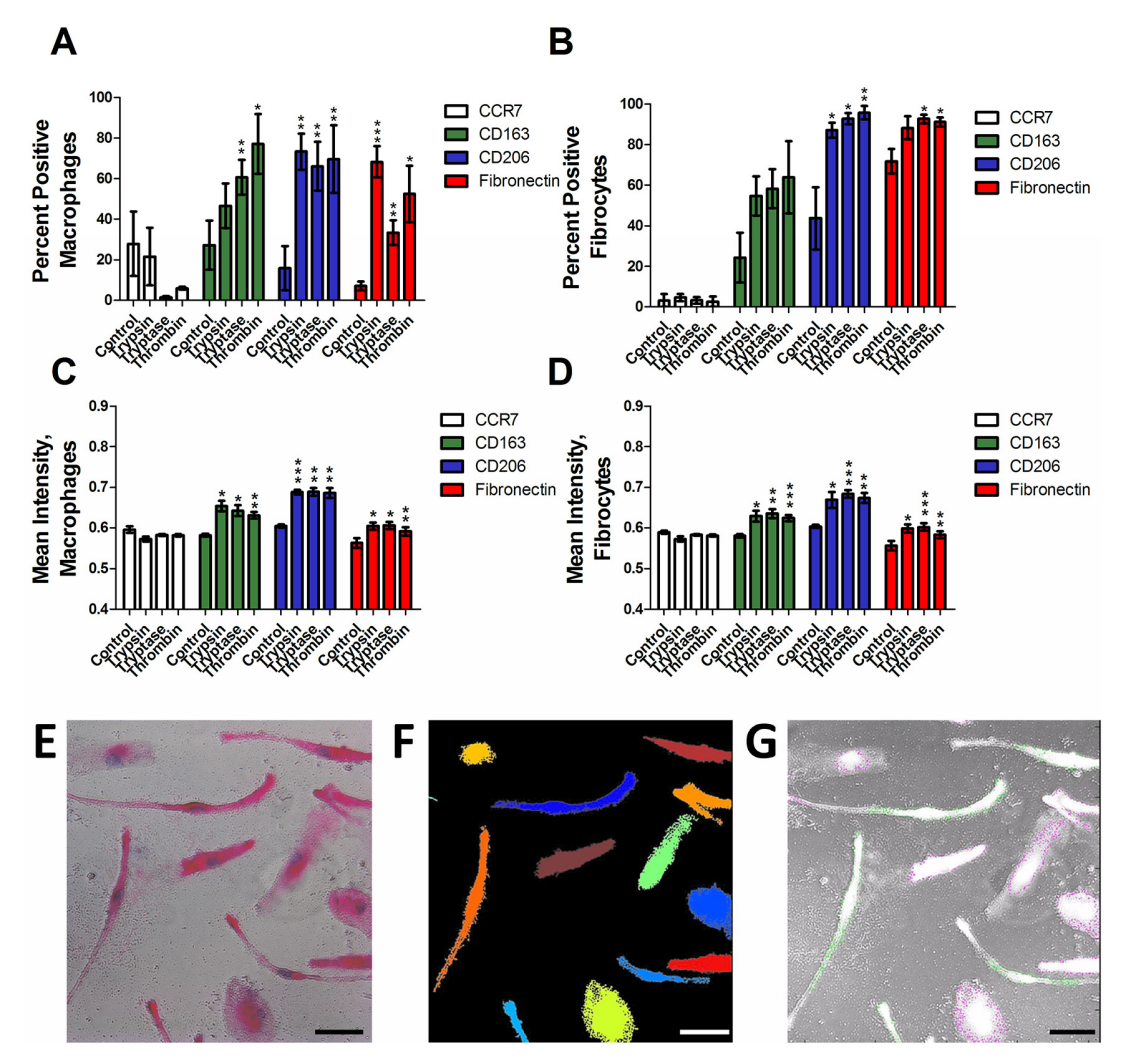
Trypsin, tryptase, and thrombin bias monocyte differentiation towards an M2a phenotype. PBMC were cultured in serum-free media in the presence or absence of trypsin, tryptase, or thrombin. Macrophages **(A, C)** and fibrocytes **(B, D)** were counted by morphology from representative fields of view. **(A)** and **(B)** were performed by eye, while **(C)** and **(D)** show analysis of staining intensity. Cells were stained for the indicated markers. Values are mean ± SEM, n = 6. * indicates p < .05, ** p < .01, and *** p < .001 compared to the no-protease control (paired two-tailed t-tests). **(E)** A representative image analyzed by CellProfiler. **(F)** The detected cells are shown as solid colors. **(G)** Cells identified as macrophages are outlined in red by the program, while cells identified as fibrocytes are outlined in green. Bars are 50 μm.

**Fig 2:**
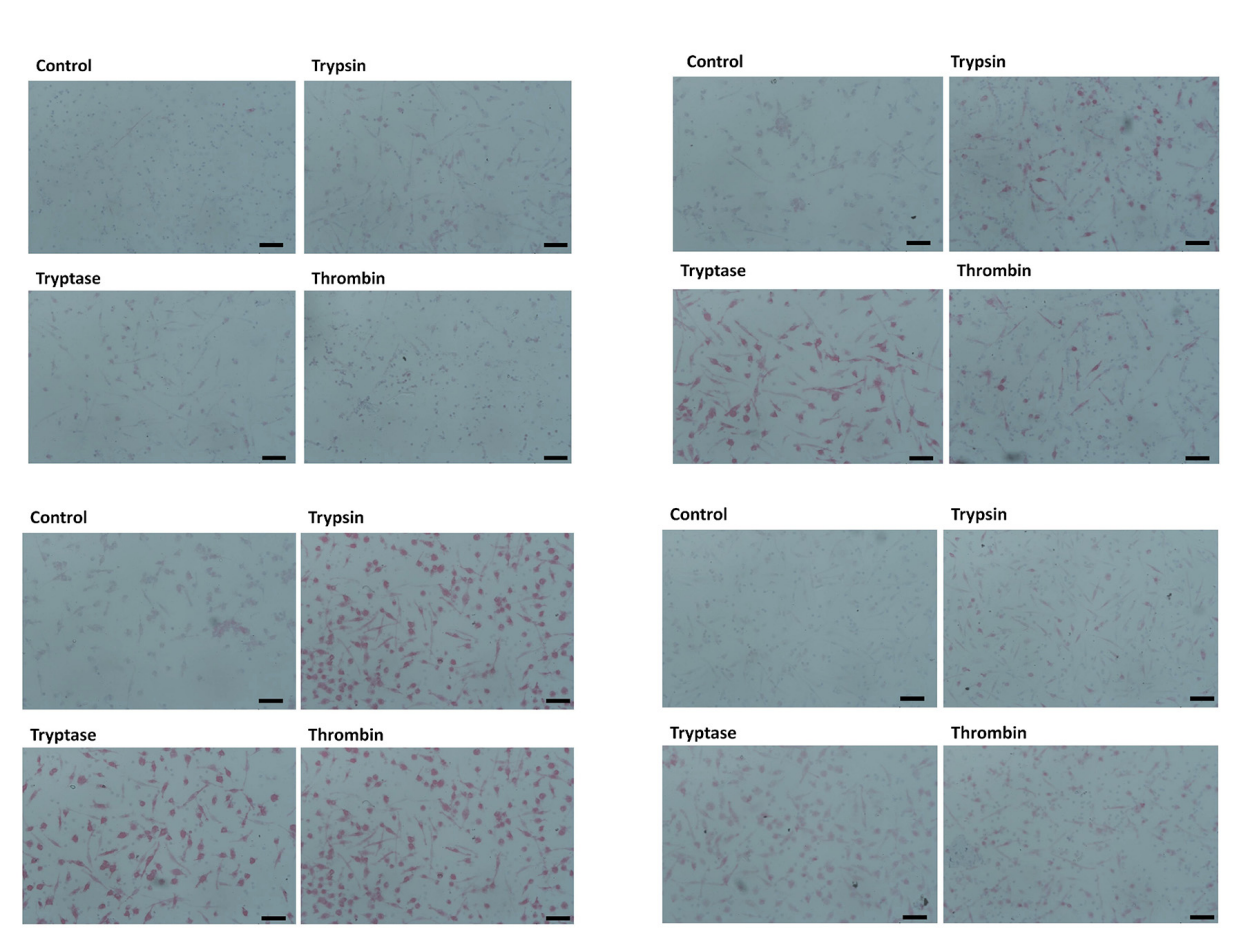
Images of PBMC cultured with proteases. Panels show representative images from slides used for Fig 1, staining for **(A)** CCR7 (mouse monoclonal clone 150503), **(B)** CD163 (mouse monoclonal clone GH1/61), **(C)** CD206 (mouse monoclonal clone 15–2), and **(D)** fibronectin. Bars are 50 μm.

To determine if trypsin, tryptase, or thrombin influence the differentiation of monocytes into macrophages, we co-incubated PBMC and proteases for five days, and examined the expression of CCR7, CD163, CD206 and fibronectin on the macrophages. We used physiological concentrations of proteases that we previously observed to potentiate fibrocyte differentiation [17, 46]. CCR7 is a marker for M1 macrophage activation [25]. No protease increased CCR7 staining (Figs 1 and 2). CD163 and CD206 are markers of Mreg and M2a macrophages [25, 37]. Tryptase and thrombin increased the percentage of CD163-positive macrophages, and all three proteases increased the percentage of CD206-positive macrophages (Figs 1 and 2). Fibronectin is a marker of M2a macrophage differentiation [38]. Each protease significantly increased the percentage of fibronectin-positive macrophages (Figs 1 and 2). In these assays, each protease also increased the percentage of fibrocytes expressing CD206 (Figs 1 and 2) and tryptase and thrombin increased the percentage of fibrocytes expressing fibronectin.

**Fig 3:**
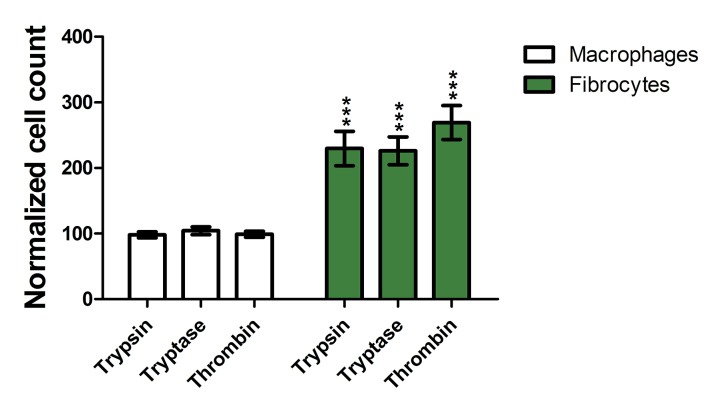
Proteases increase fibrocyte numbers when added to PBMC. Cellprofiler counts of fibrocytes and macrophages from slides used for Fig 1 were normalized as a percent of controls. Values are mean ± SEM, n = 6. *** indicates p < .001 compared to the no-protease control (unpaired two-tailed t-tests).

To determine if protease treatment changed the staining intensity of the macrophage marker immunohistochemistry, stained slides were further analyzed by image analysis that identified cells as macrophages, fibrocytes, or other cells and then measured the mean staining intensity of each cell (Fig 1). Comparison to a manual assessment indicated that image analysis correctly identified 85% of fibrocytes, identified 2% of fibrocytes as macrophages, and did not detect 13% of the fibrocytes. Similarly, the image analysis correctly identified 86% of macrophages, identified 5% of macrophages as fibrocytes, and did not detect 9% of the macrophages. As expected, the image analysis also detected that the proteases increased the number of fibrocytes (Fig 3). Trypsin, tryptase, and thrombin did not increase the staining intensity of CCR7, and increased the mean staining intensity of CD163, CD206, and fibronectin on both macrophages and fibrocytes (Fig 1). Together, the data indicate that as determined by staining for M1, M2, and M2a markers, the proteases biased monocyte differentiation towards an M2a phenotype.

**Fig 4:**
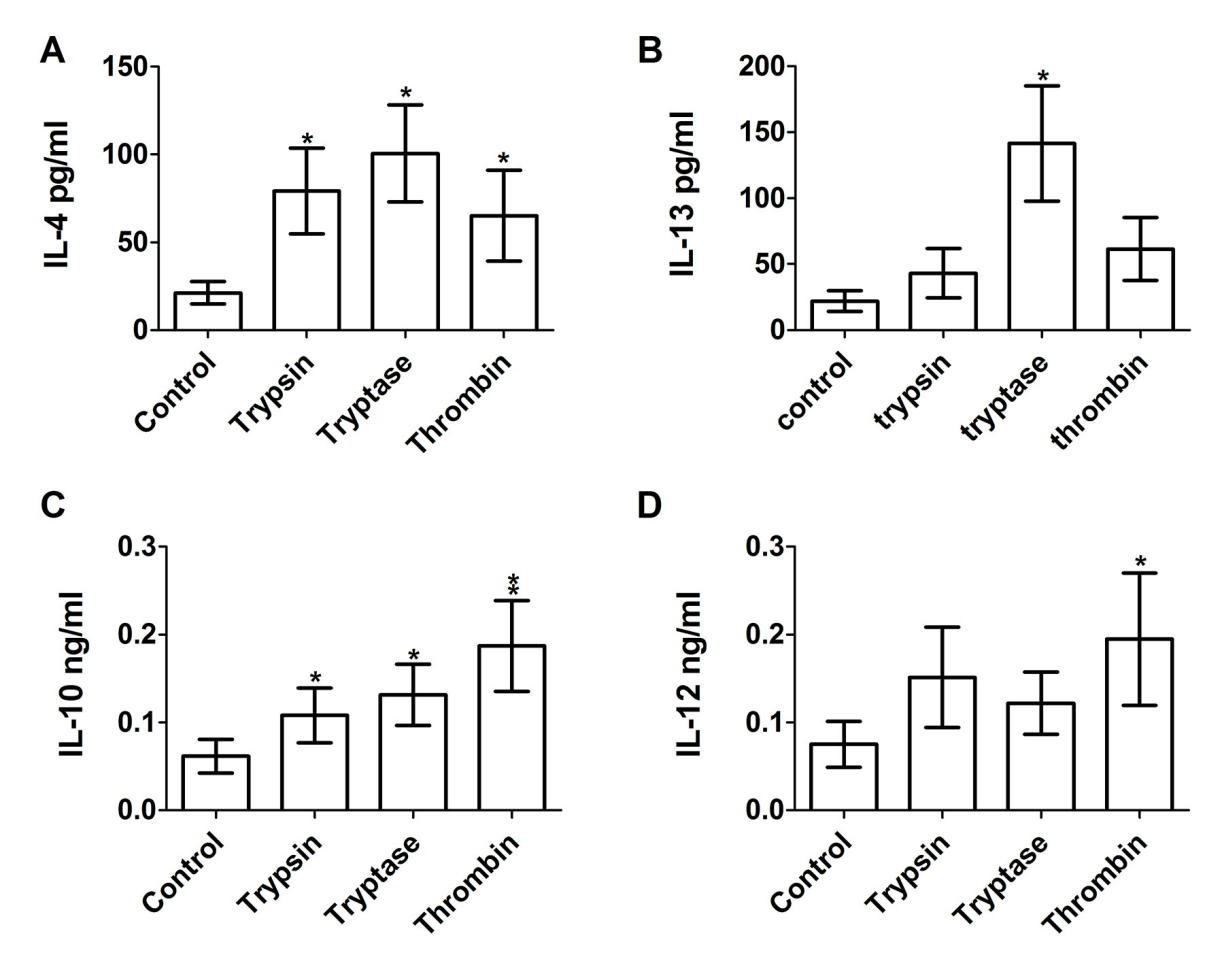
The effect of proteases on extracellular cytokine accumulation from cultures of PBMC. PBMC were cultured as in Fig 1, and after 5 days conditioned media were analyzed by ELISA for **(A)** IL-4, **(B)** IL-13, **(C)** IL-10, and **(D)** IL-12. Values are mean ± SEM, n = 24 for (A), (C), and (D), and n = 6 for (B). * indicates p < .05 and ** p < .01 compared to the no-protease control (paired two-tailed t-tests).

To determine if proteases also affect extracellular cytokine accumulation by cultured macrophages, conditioned media from human PBMC cultured for five days with 12.5 ng/ml of trypsin, tryptase, or thrombin were assayed by ELISA for IL-4, IL-13, IL-10, and IL-12. Trypsin, but not tryptase or thrombin, is predicted to cleave IL-4, IL-13, IL-10, and IL-12 (ExPASy peptide cutter). Compared to conditioned media from untreated control cells, trypsin, tryptase, and thrombin increased IL-4 and IL-10 accumulation in PBMC conditioned media, and tryptase increased IL-13 accumulation (Fig 4). IL-4 and IL-13 are secreted by and promote M2a macrophages differentiation [47]. While IL-4 can also be secreted by other cells such as basophils [48], the observed increase in IL-4 is in agreement with our observation that the proteases promote M2a macrophages. Thrombin increased IL-12 accumulation (Fig 4). The data thus suggest that trypsin, tryptase and thrombin bias monocytes towards an M2a phenotype (high levels of IL-4 and IL-13, moderate levels of IL-10 and IL-12).

### Trypsin, tryptase, and thrombin potentiate the differentiation of M2 macrophages into M2a macrophages

**Fig 5:**
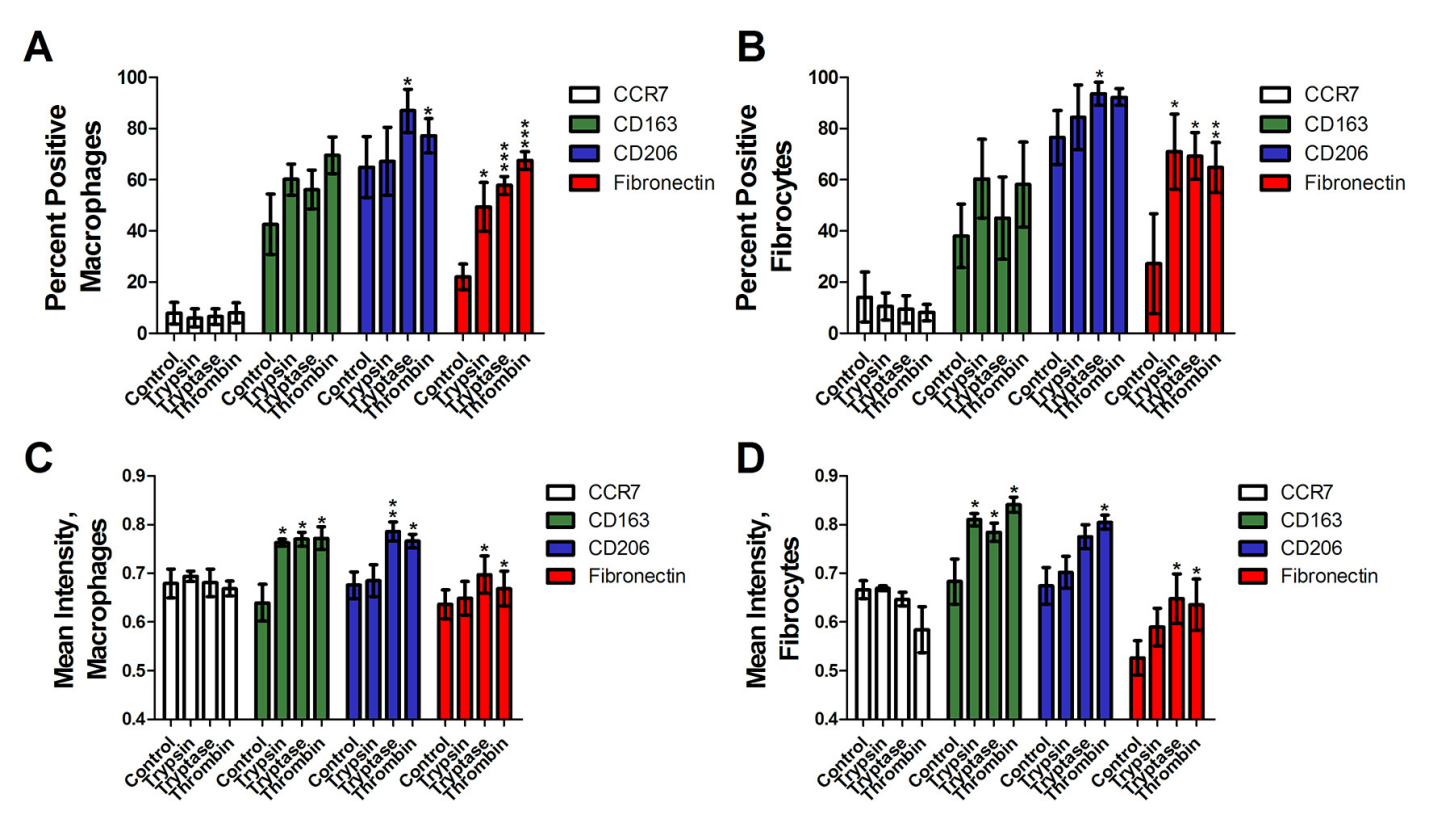
Trypsin, tryptase, and thrombin bias M2 macrophage differentiation towards an M2a phenotype. PBMC were cultured with MCSF for 7 days to generate M2 macrophages, after which the media was removed and proteases were added to the PBMC for 2 days. Macrophages **(A, C)** and fibrocytes **(B, D)** were counted by morphology from representative fields of view. **(A)** and **(B)** were performed by eye, while **(C)** and **(D)** show analysis of staining intensity. Cells were stained for the indicated markers. Values are mean ± SEM, n = 6. * indicates p < .05, ** p < .01, and *** p < .001 compared to the no-protease control (unpaired two-tailed t-tests).

**Fig 6:**
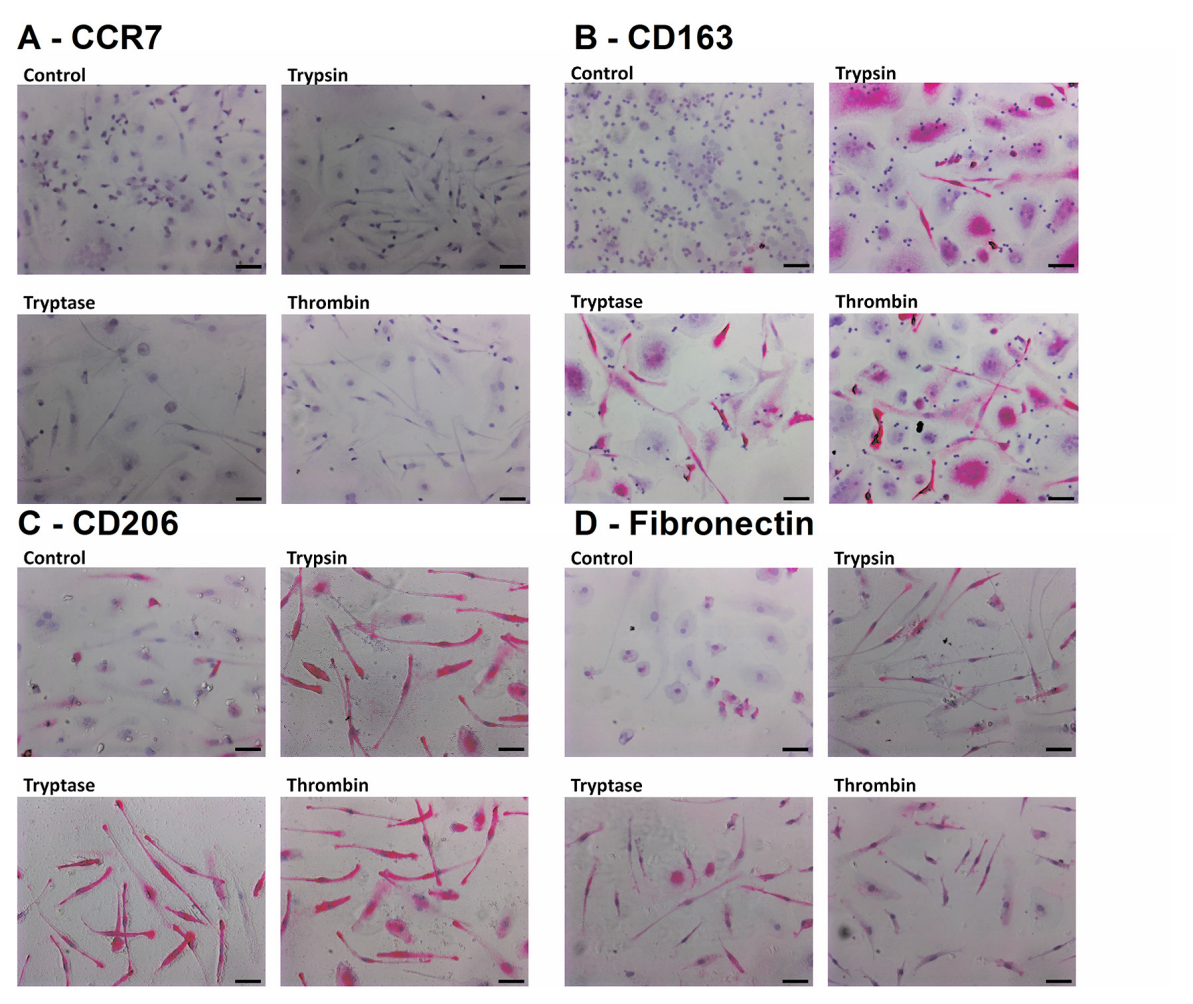
Images of cultures containing M2-biased macrophages subsequently cultured with proteases. Panels show representative images from slides used for Fig 5, staining for **(A)** CCR7 (mouse monoclonal clone 150503), **(B)** CD163 (mouse monoclonal clone GH1/61), **(C)** CD206 (mouse monoclonal clone 15–2), and **(D)** fibronectin. Bars are 50 μm.

**Fig 7:**
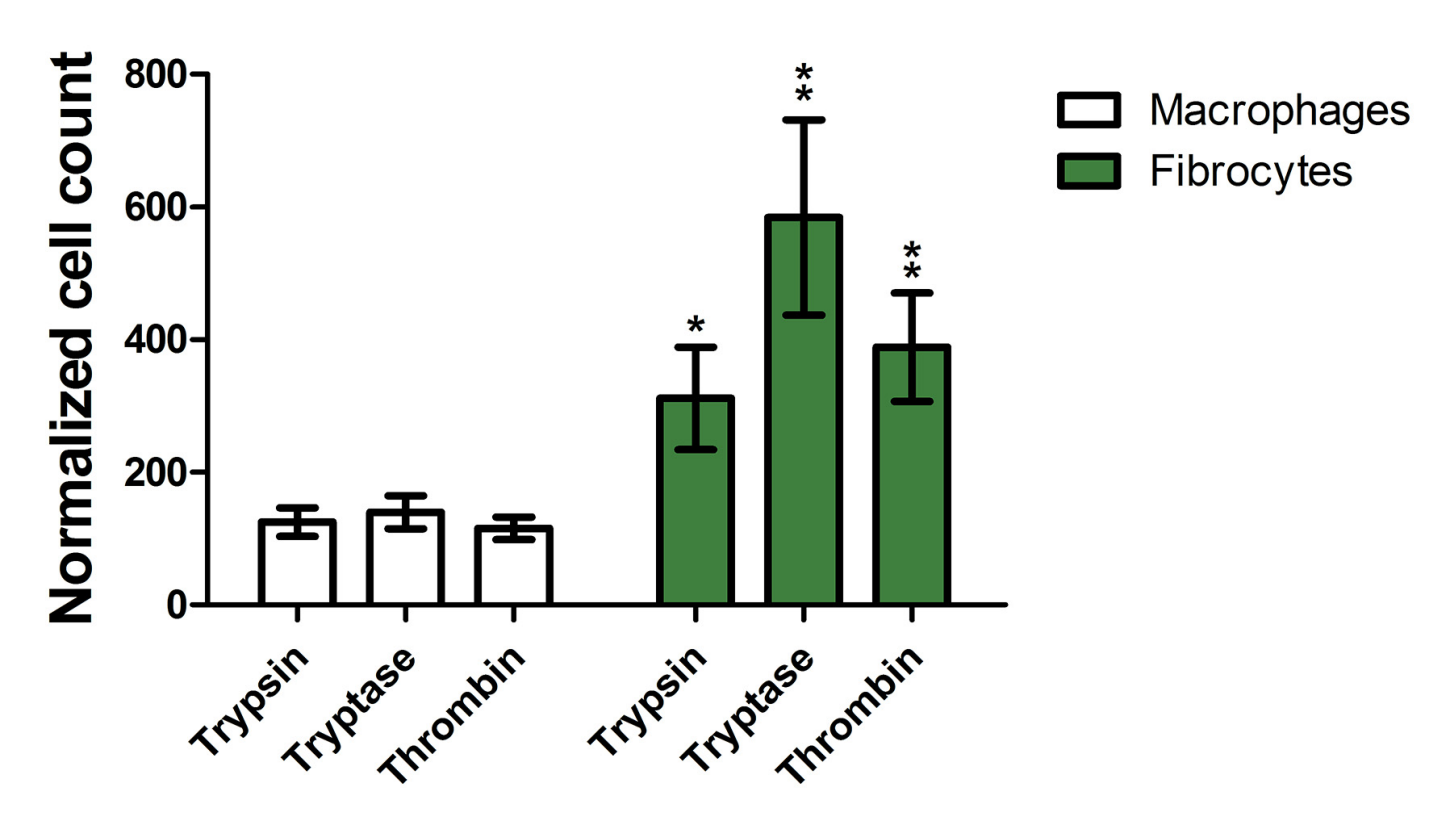
Proteases increase fibrocyte numbers when added to cultures containing M2-biased macrophages. Cellprofiler counts of fibrocytes and macrophages from slides used for Fig 5 were normalized as a percent of controls. Values are mean ± SEM, n = 6. * indicates p < .05 and ** p < .01 compared to the no-protease control (paired two-tailed t-tests).

M2a macrophages can differentiate not only from unpolarized monocytes, but also from other macrophage subsets [49]. M2 macrophages are associated with decreased inflammation and increased tissue repair [26, 30]. To determine if proteases can potentiate the differentiation of M2a macrophages from M2 macrophages, we biased unpolarized monocytes towards an M2 phenotype, as previously described [42], after which we added proteases to the macrophage population for two days, and stained for macrophage markers. Compared to the macrophage controls in Fig 1, adding MCSF significantly increased the number and intensity of CD206 stained cells (p < 0.05), and significantly decreased the intensity of CCR7 staining (p < 0.05) by an unpaired two-tailed t-test. This indicates that the GMCSF is affecting the macrophages as expected [42]. Trypsin, tryptase, and thrombin had no significant effect on the percentage of macrophages staining for the M1 marker CCR7 or the M2 marker CD163, both tryptase and thrombin significantly increased the percentage of CD206-positive macrophages, and all three proteases increased the percentage of fibronectin-positive macrophages (Figs 5 and 6). In this assay, tryptase increased the percentage of fibrocytes that were positive for CD206, and trypsin, tryptase, and thrombin increased the percentage of fibrocytes that were positive for fibronectin (Figs 5 and 6). All three proteases had no significant effect on CCR7 staining intensity, increased the staining intensity of CD163 on macrophages, and tryptase and thrombin increased the staining intensities of CD206 and fibronectin on macrophages (Fig 5). CellProfiler also detected that the proteases increased the number of fibrocytes when added to the M2-biased PBMC population (Fig 7). All three proteases increased the staining intensity of CD163 on fibrocytes, thrombin increased the CD206 staining intensity, and tryptase and thrombin increased the fibronectin staining intensity (Fig 5). Together, the data indicate that as determined by staining for M1, M2, and M2a markers, the proteases biased M2 macrophage polarization towards an M2a phenotype.

**Fig 8:**
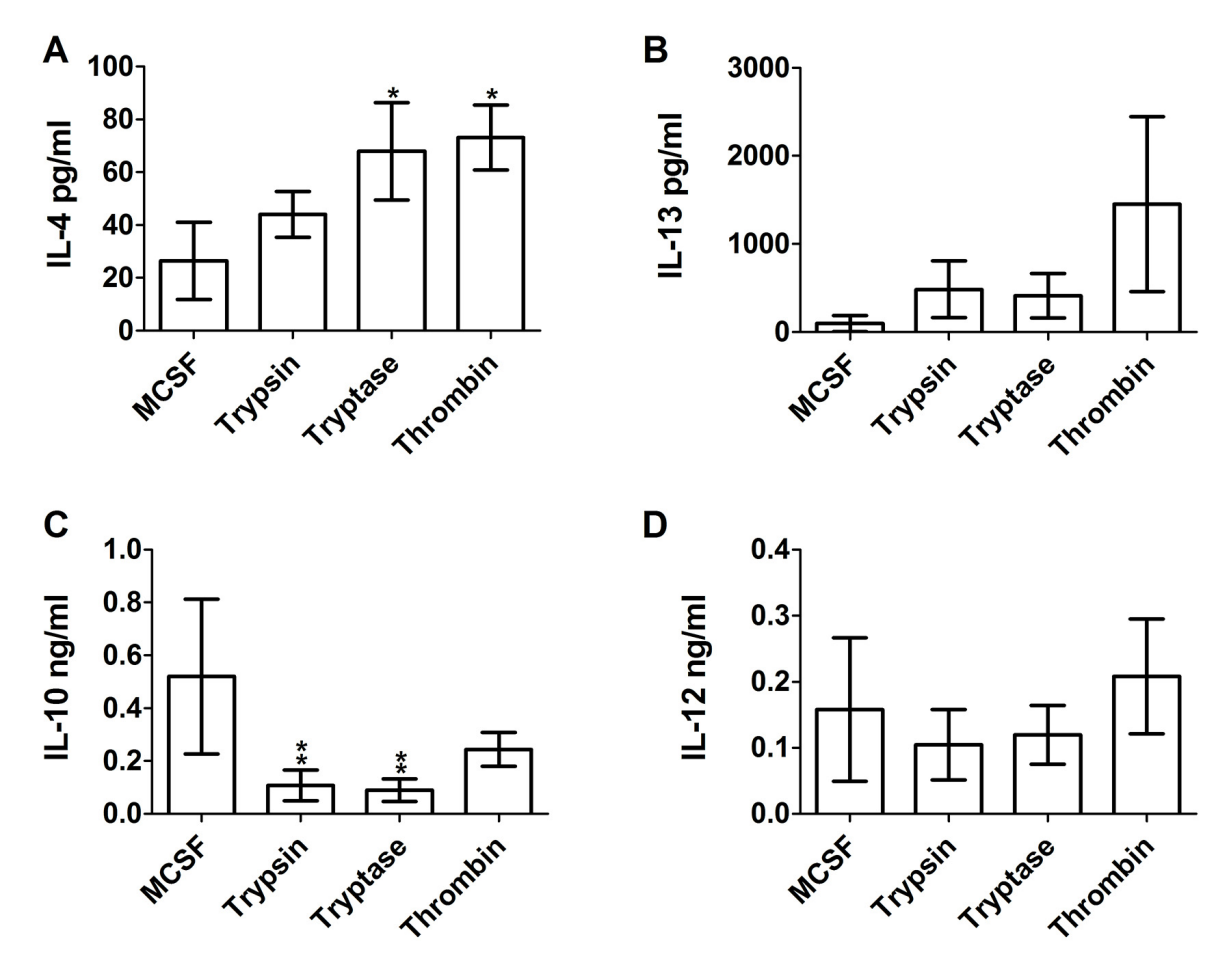
The effect of proteases on extracellular cytokine accumulation from cultures containing M2-biased macrophages. PBMC were cultured as in Fig 5, and after 2 days of protease treatment, conditioned media were analyzed by ELISA for **(A)** IL-4, **(B)** IL-13, **(C)** IL-10, and **(D)** IL-12. Values are mean ± SEM, n = 6. * indicates p < .05 and ** p < .01 compared to the no-protease control (paired two-tailed t-tests).

To determine if proteases also affect extracellular cytokine accumulation by cultured M2 macrophages, conditioned media from M2 macrophages cultured for two days with trypsin, tryptase, or thrombin were assayed by ELISA for IL-4, IL-13, IL-10, and IL-12. Tryptase and thrombin increased IL-4 accumulation (Fig 8). Trypsin and tryptase decreased IL-10 (Fig 8). No protease significantly altered IL-12 or IL-13 concentrations (Fig 8). The data thus suggest that the proteases may bias M2 macrophages towards an M2a phenotype by either decreasing the concentration of the anti-inflammatory cytokine IL-10 or increasing the concentration of the profibrotic cytokine IL-4.

### Trypsin, tryptase, and thrombin potentiate the differentiation of M1 macrophages into M2a macrophages

**Fig 9:**
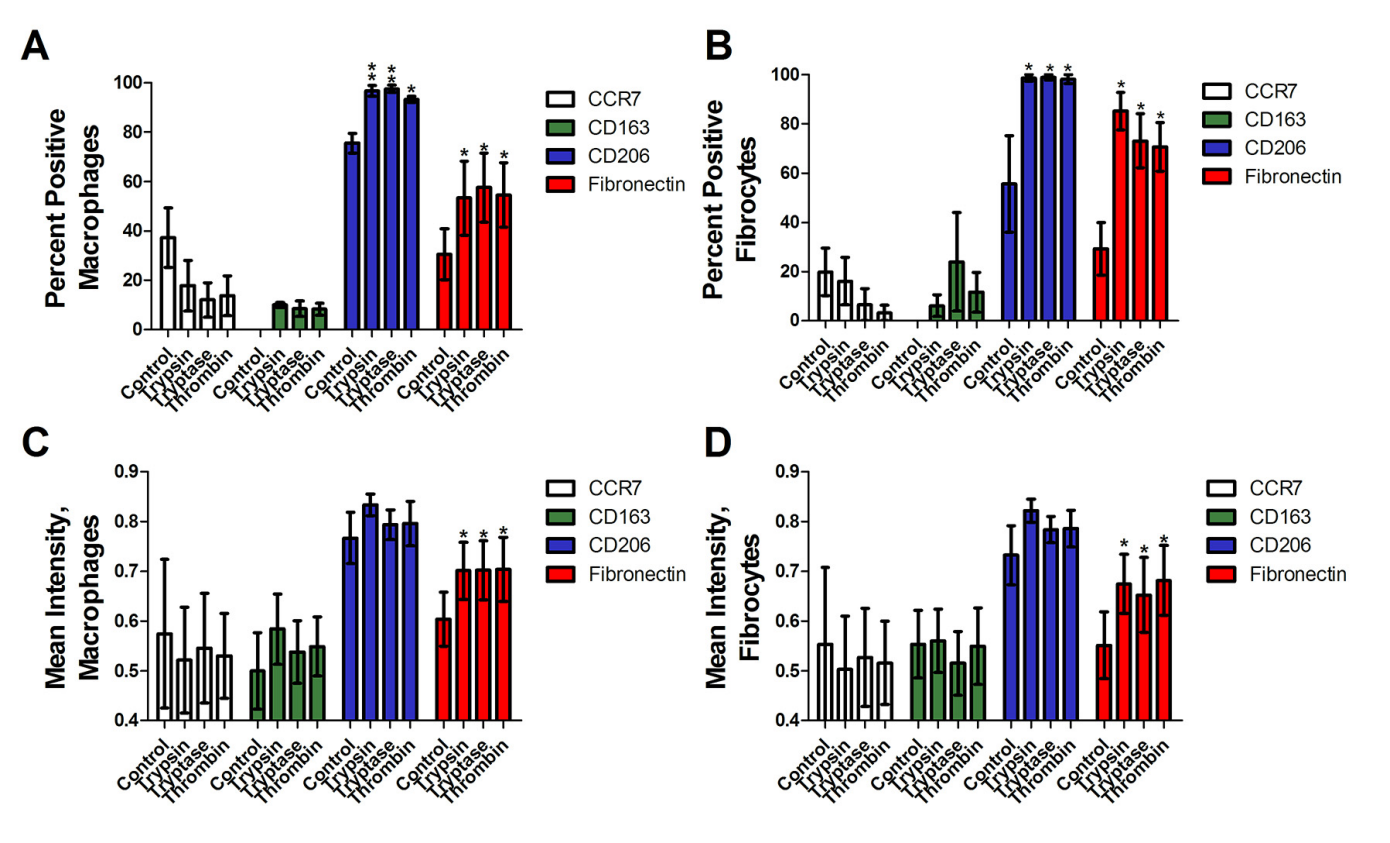
Trypsin, tryptase, and thrombin bias M1 macrophage differentiation towards an M2a phenotype. PBMC were cultured with GMCSF for 7 days to generate M2 macrophages, after which the media was removed and proteases were added to the PBMC for 2 days. Macrophages **(A, C)** and fibrocytes **(B, D)** were counted by morphology from representative fields of view. **(A)** and **(B)** were performed by eye, while **(C)** and **(D)** show analysis of staining intensity. Cells were stained for the indicated markers. Values are mean ± SEM, n = 6. * indicates p < .05 and ** p < .01 compared to the no-protease control (unpaired two-tailed t-tests).

**Fig 10:**
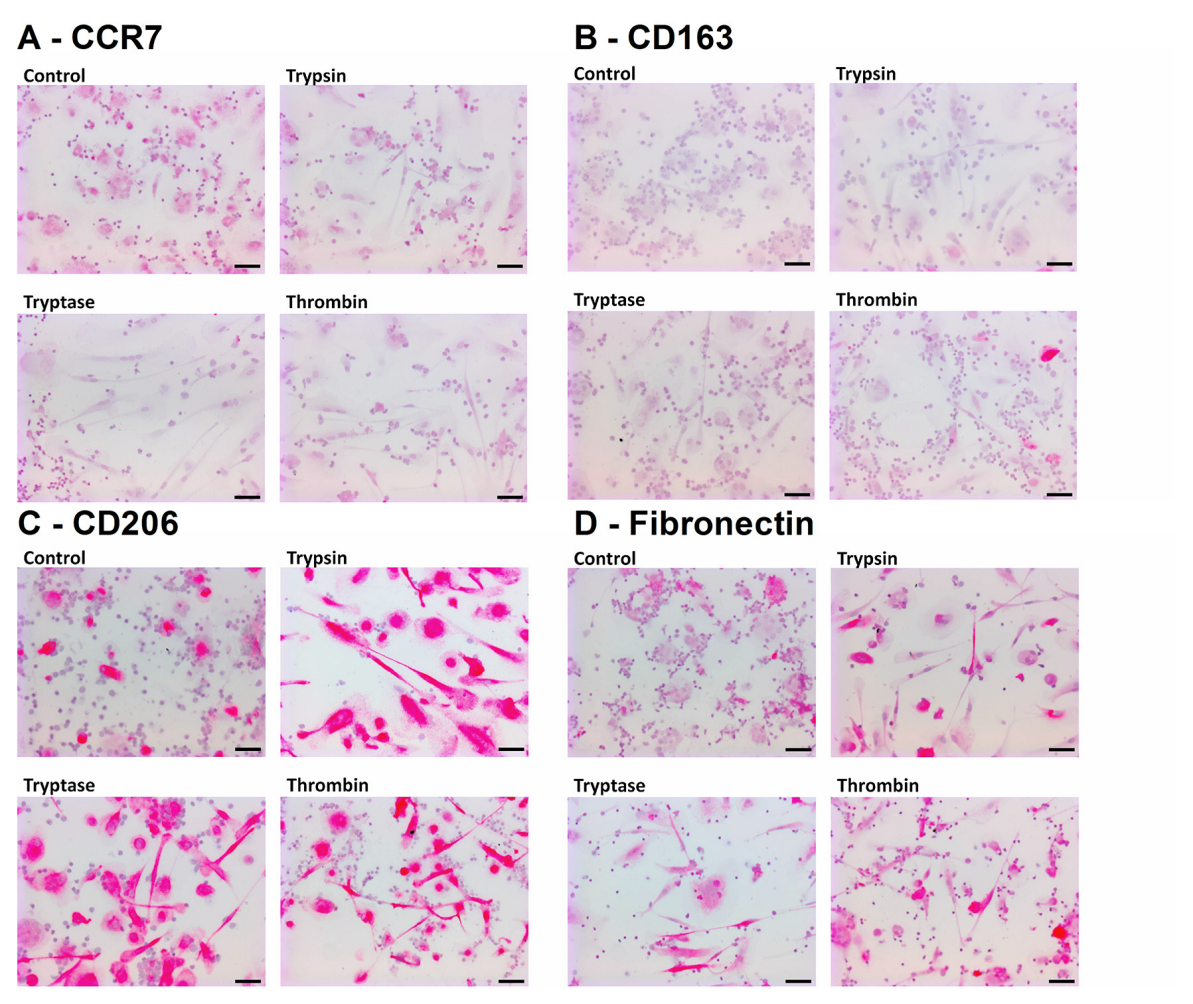
Images of cultures containing M1-biased macrophages subsequently cultured with proteases. Panels show representative images from slides used for Fig 9, staining for **(A)** CCR7, **(B)** CD163, **(C)** CD206, and **(D)** fibronectin. Bars are 50 μm.

**Fig 11:**
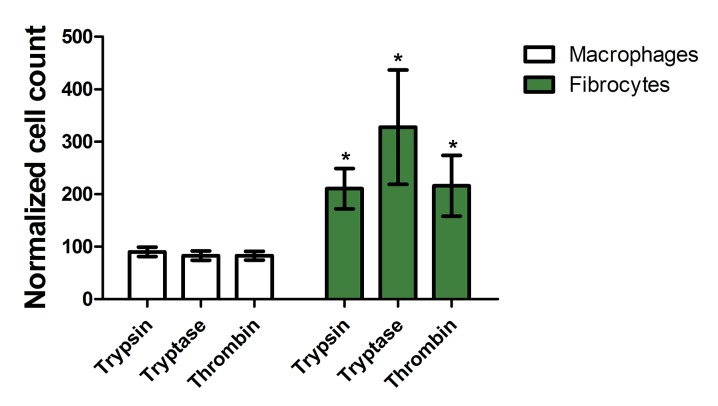
Proteases increase fibrocyte numbers when added to cultures containing M1-biased macrophages. Cellprofiler counts of fibrocytes and macrophages from slides used for Fig 9 were normalized as a percent of controls. Values are mean ± SEM, n = 6. * indicates p < .05 compared to the no-protease control (paired two-tailed t-tests).

M1 macrophages are associated with inflammatory immune responses to pathogens like bacteria and viruses [25]. To determine if proteases could bias M1 macrophages towards an M2a phenotype, we biased monocytes towards an M1 phenotype [42], after which we added proteases to the macrophage population for two days, and stained for macrophage markers. Compared to the macrophage controls in Fig 1, adding GMCSF significantly increased the number and intensity of CD206 stained cells (p < 0.05), and significantly decreased the number of CD163 positive cells (p < 0.05) by an unpaired two-tailed t-test. Compared to the macrophages polarized by MCSF, polarizing the macrophages with GMCSF significantly increased CCR7 and decreased CD163 staining (p < 0.05) by an unpaired two-tailed t-test. Compared to no MCSF or GMCSF treatment (Fig 1), both MCSF and GMCSF increased CD206 staining, as observed by other groups [42]. This indicates that the GMCSF is affecting the macrophages as expected [42, 50]. None of the proteases significantly affected CCR7 or CD163 staining, but all three increased the percentage of macrophages staining for CD206 and fibronectin, and all three increased fibronectin intensity staining on macrophages (Figs 9 and 10). CellProfiler also detected that the proteases increased the number of fibrocytes when added to the M1-biased PBMC population (Fig 11). Each protease increased CD206 and fibronectin staining on fibrocytes, and each protease increased fibronectin staining intensity staining on fibrocytes (Figs 9 and 10). Together, the data indicate that as determined by staining for M1, M2, and M2a markers, the proteases biased M1 macrophage polarization towards an M2a phenotype.

**Fig 12:**
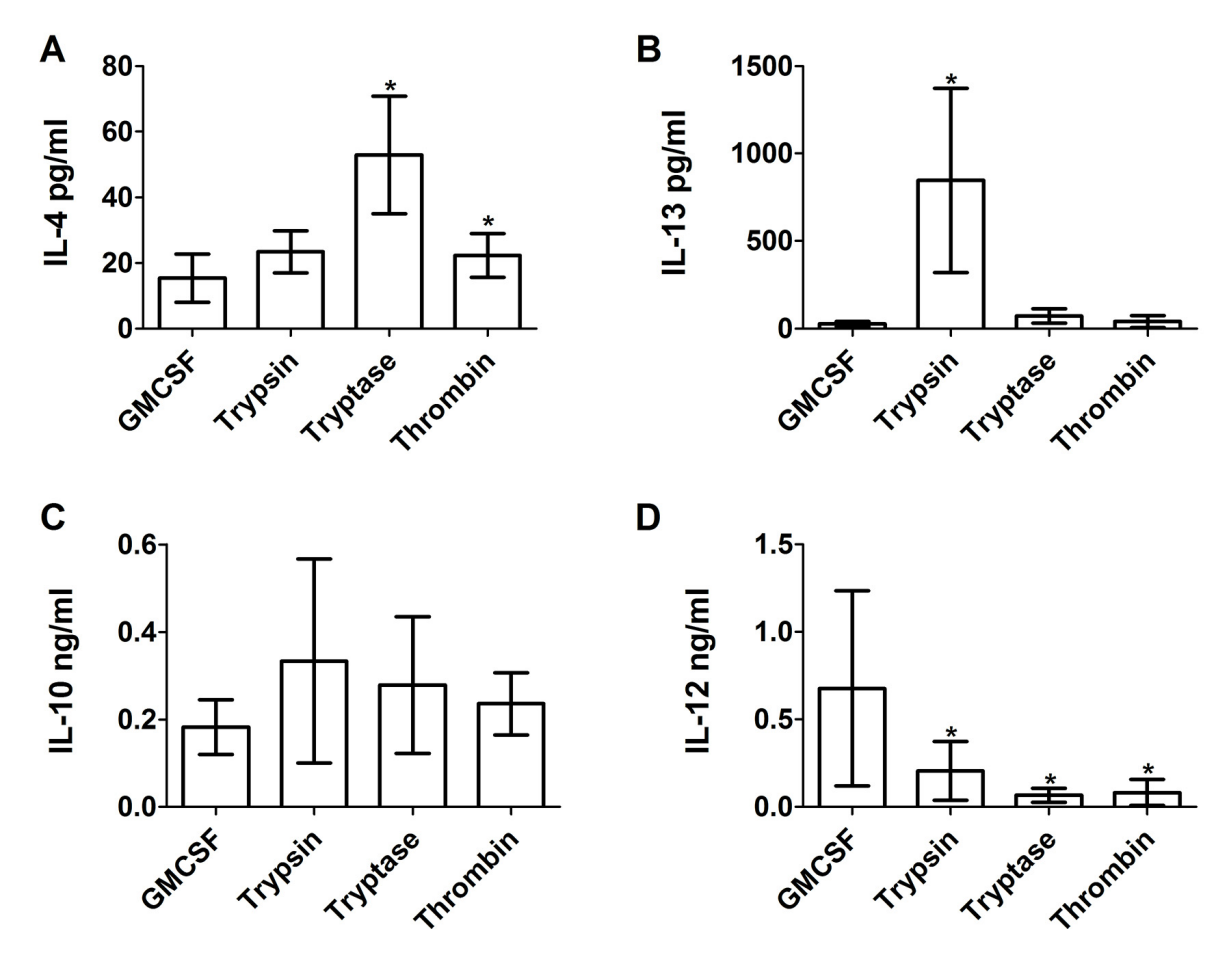
The effect of proteases on extracellular cytokine accumulation from cultures containing M1-biased macrophages. PBMC were cultured as in Fig 9, and after 2 days of protease treatment, conditioned media were analyzed by ELISA for **(A)** IL-4, **(B)** IL-13, **(C)** IL-10, and **(D)** IL-12. Values are mean ± SEM, n = 6. * indicates p < .05 compared to the no-protease control (paired two-tailed t-tests).

To determine if proteases also affect extracellular cytokine accumulation by cultured M1 macrophages, conditioned media from human M1 macrophages cultured for two days with trypsin, tryptase, or thrombin were assayed by ELISA for IL-4, IL-13, IL-10, and IL-12. Tryptase and thrombin increased IL-4 accumulation, while trypsin increased IL-13 accumulation (Fig 12), no protease significantly affected IL-10, and all three proteases decreased IL-12 accumulation (Fig 12). The data thus indicate that as determined by cytokine accumulation, trypsin, tryptase and thrombin polarize M1 macrophages towards an M2a phenotype.

**Fig 13:**
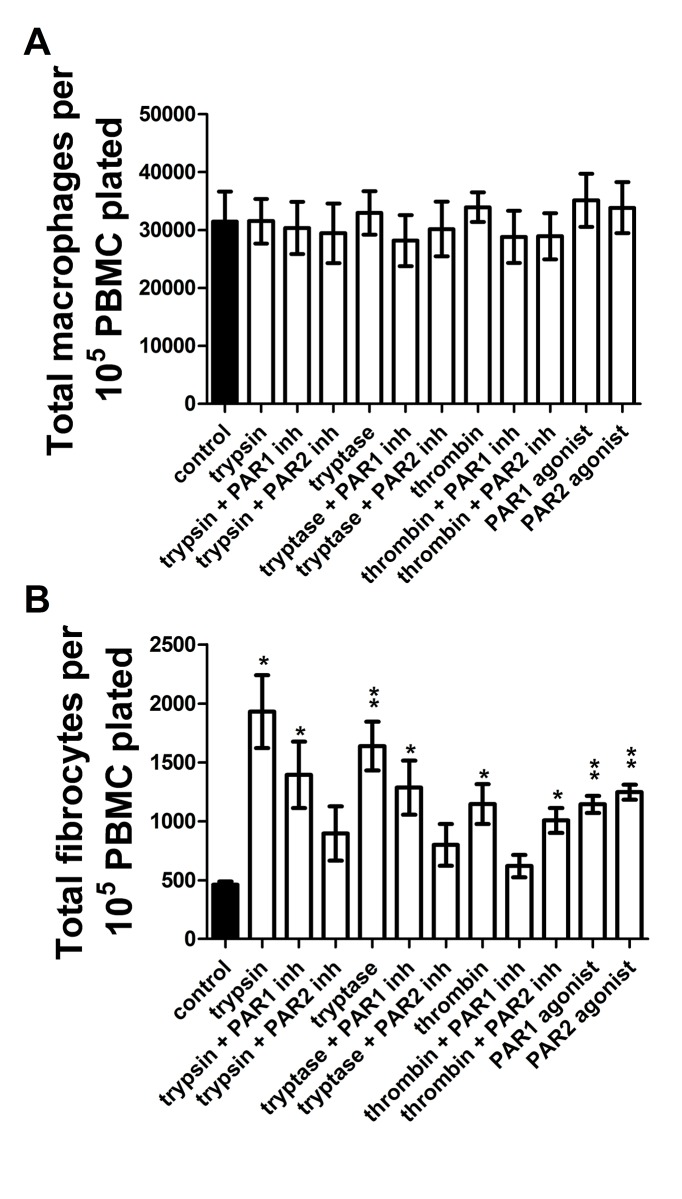
Proteases potentiate fibrocyte differentiation through activation of PAR-1 and PAR-2 receptors. PBMC were cultured as in Fig 1, and after five days of treatment by PAR agonists, proteases, or proteases in the presence of PAR-1 or PAR-2 inhibitors, were analyzed as in Fig 1. **(A)** macrophage and **(B)** fibrocyte counts. Values are mean ± SEM, n = 6. * indicates p < .05 and ** indicates p < .01 compared to the no-protease control (paired two-tailed t-tests).

**Fig 14:**
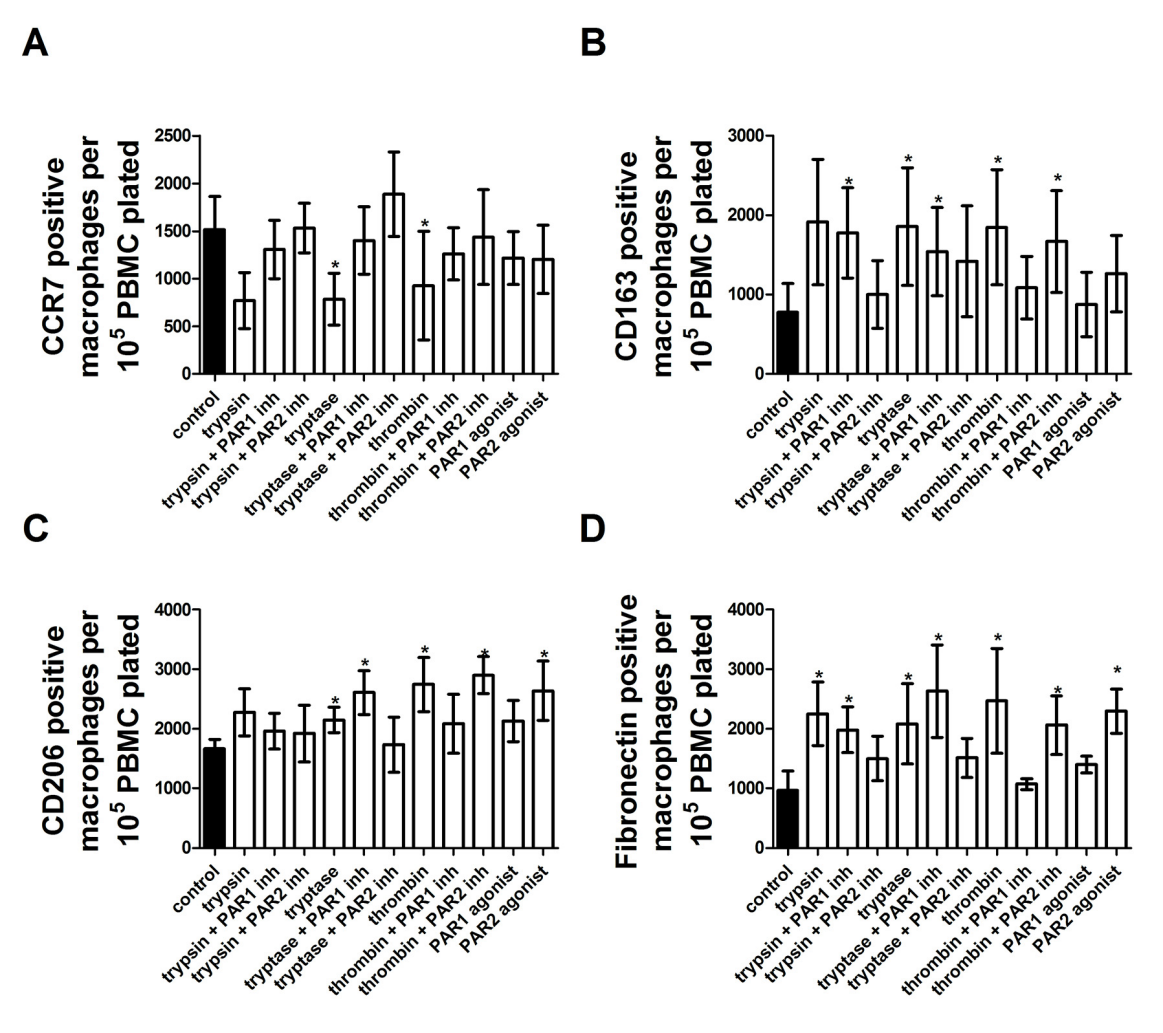
Proteases induce macrophage polarization through activation of PAR-1 and PAR-2. PBMC were cultured as in Fig 1, and after five days of treatment by PAR agonists, proteases, or proteases in the presence of PAR-1 or PAR-2 inhibitors, were analyzed as in Fig 1A. **(A)** CCR7 positive macrophages, **(B)** CD163 positive macrophages, **(C)** CD206 positive macrophages, and **(D)** fibronectin positive macrophages. Values are mean ± SEM, n = 6. * indicates p < .05 compared to the no-protease control (paired two-tailed t-tests).

Proteases induce fibrocyte differentiation through activation of protease-activated receptors -1 and -2 (PARs -1 and -2) [41]. To determine if proteases induce macrophage differentiation through activation of PAR-1 and/or PAR-2, PAR-1 and PAR-2 agonists were added to PBMC for five days. PAR-1 and PAR-2 inhibitors were also added to PBMC in the presence of trypsin, tryptase, or thrombin. The resulting populations were stained for CCR7, CD163, CD206, and fibronectin. Each of trypsin, tryptase, thrombin, PAR-1 agonist, and PAR-2 agonist potentiated fibrocyte differentiation, while inhibitors of PAR-1 signaling blocked thrombin-induced fibrocyte differentiation and inhibitors of PAR-2 signaling blocked trypsin and tryptase-induced fibrocyte differentiation (Fig 13). No protease or PAR agonist induced a significantly different number of macrophages (Fig 13). For macrophages, each of trypsin, tryptase, and thrombin increased fibronectin staining. PAR-1, but not PAR-2, inhibitor returned fibronectin staining to control levels in the presence of thrombin. PAR-2 inhibitor returned fibronectin staining to control levels in the presence of trypsin and tryptase. PAR-1 inhibitor did not diminish fibronectin staining in the presence of tryptase, but did diminish staining in the presence of trypsin. PAR-2 agonist, but not PAR-1 agonist, increased fibronectin staining (Fig 14D).

Tryptase and thrombin increased both CD163 and CD206 staining, while trypsin did not increase either. PAR-1, but not PAR-2, inhibitor returned CD163 and CD206 staining to control levels in the presence of thrombin. PAR-2, but not PAR-1, inhibitor returned CD163 and CD206 staining to control levels in the presence of tryptase. PAR1 and PAR2 agonist did not increase either CD163 or CD206 staining (Fig 14B and 14C).

Tryptase and thrombin decreased CCR7 staining, while both PAR-1 and PAR-2 inhibitors returned CCR7 staining to control levels in the presence of tryptase and thrombin. Neither trypsin, PAR-1 agonist, or PAR-2 agonist effected CCR7 staining (Fig 14A).

**Fig 15:**
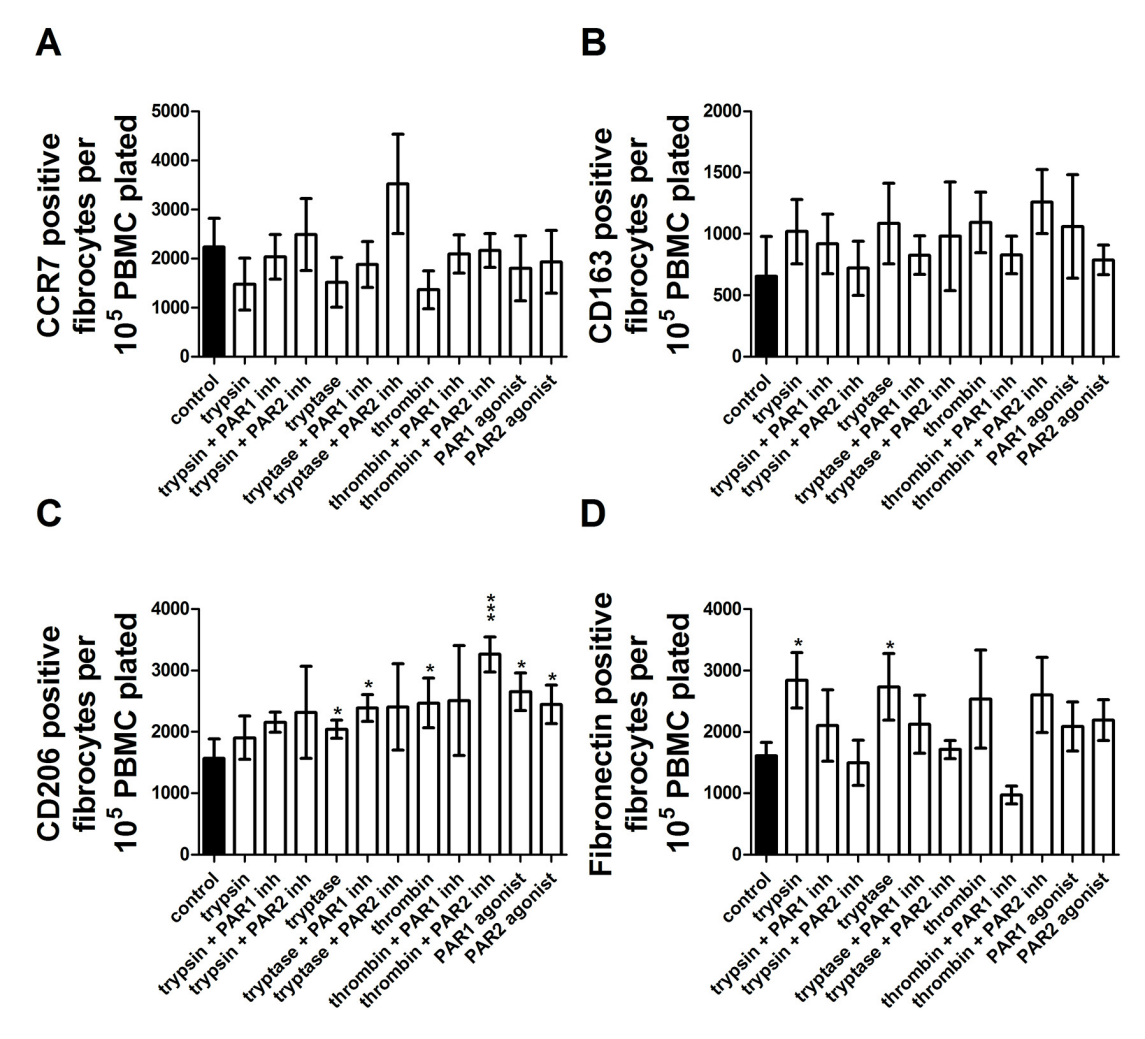
Proteases induce changes in fibrocyte marker expression through activation of PAR-1 and PAR-2. PBMC were cultured as in Fig 1, and after five days of treatment by PAR agonists, proteases, or proteases in the presence of PAR-1 or PAR-2 inhibitors, were analyzed as in Fig 1B. **(A)** CCR7 positive fibrocytes, **(B)** CD163 positive fibrocytes, **(C)** CD206 positive fibrocytes, and **(D)** fibronectin positive fibrocytes. Values are mean ± SEM, n = 6. * indicates p < .05 and *** indicates p < .001 compared to the no-protease control (paired two-tailed t-tests).

For fibrocytes, no treatment increased or decreased CCR7 or CD163 staining. Tryptase and thrombin increased CD206 staining, along with PAR-1 and PAR-2 agonists. PAR-1, but not PAR-2, inhibitor returned CD206 staining to control levels in the presence of thrombin. PAR-2, but not PAR-1, inhibitor returned CD206 to control levels in the presence of tryptase (Fig 15C). Both trypsin and tryptase increased fibronectin staining, but not other condition increased fibronectin staining for fibrocytes (Fig 15D).

**Fig 16:**
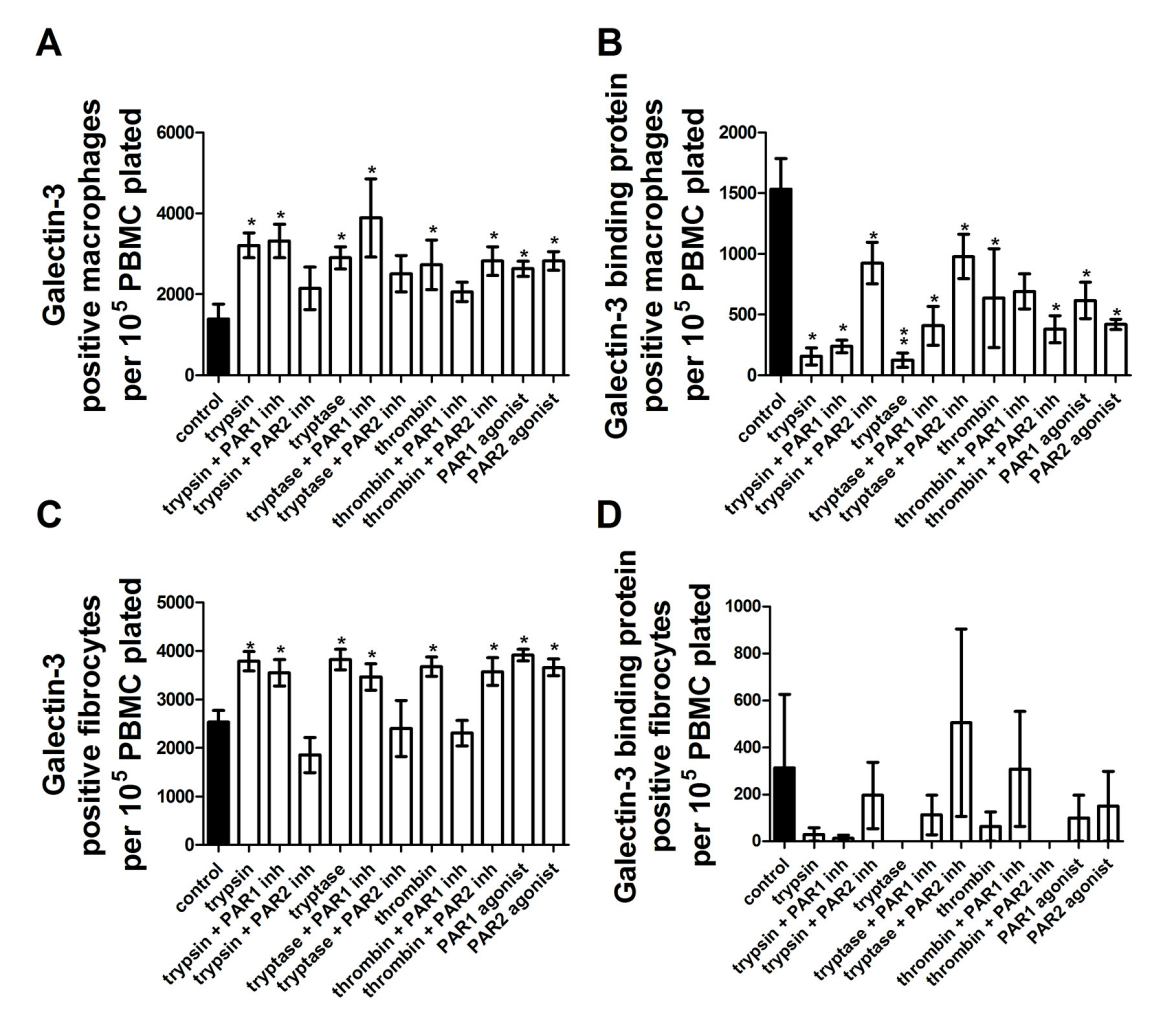
Galectin-3 and galectin-3 binding protein are markers of macrophage polarization and fibrocyte differentiation. PBMC were cultured as in Fig 1, and after five days of treatment by PAR agonists, proteases, or proteases in the presence of PAR-1 or PAR-2 inhibitors, were analyzed as in Fig 1A (for (A) and (B)) and Fig 1B (for (C) and (D)). **(A)** Galectin-3 positive macrophages, **(B)** galectin-3 binding protein positive macrophages, **(C)** galectin-3 positive fibrocytes, **(D)** galectin-3 binding protein positive fibrocytes. Values are mean ± SEM, n = 6. * indicates p < .05 and ** indicates p < .01 compared to the no-protease control (paired two-tailed t-tests).

Galectin-3 potentiates fibrocyte differentiation, and galectin-3 binding protein inhibits fibrocyte differentiation [51]. To determine if galectin-3 and galectin-3 binding protein might be affected by PAR-1 or PAR-2 activation, we stained PBMC for galectin-3 and galectin-3 binding protein in the presence of proteases, PAR agonists and PAR inhibitors. Galectin-3 and galectin-3 binding protein proved to be excellent markers of fibrocyte differentiation, and were more responsive to the addition of protease, PAR inhibitors or PAR than CCR7, CD163, CD206 and fibronectin. For macrophages, each of trypsin, tryptase, thrombin, PAR-1 agonist, and PAR-2 agonist increased galectin-3 staining. PAR-1, but not PAR-2, inhibitor reduced galectin-3 staining to control levels in the presence of thrombin, while PAR-2, but not PAR-1, inhibitor reduced galectin-3 staining to control levels in the presence of trypsin and tryptase (Fig 16A). Every condition, even in the presence of PAR-1 or PAR-2 inhibitor, reduced galectin-3 binding protein staining (Fig 16B). For fibrocytes, each of trypsin, tryptase, thrombin, PAR-1 agonist, and PAR-2 agonist increased galectin-3 staining. PAR-1, but not PAR-2, inhibitor reduced galectin-3 staining to control levels in the presence of thrombin, while PAR-2, but not PAR-1, inhibitor reduced galectin-3 staining to control levels in the presence of trypsin and tryptase (Fig 16C). Very few fibrocytes were positive for galectin-3 binding protein under any condition (Fig 16D). These results suggest that galectin-3 and galectin-3 binding partner may be useful markers for macrophage polarization.

**Fig 17:**
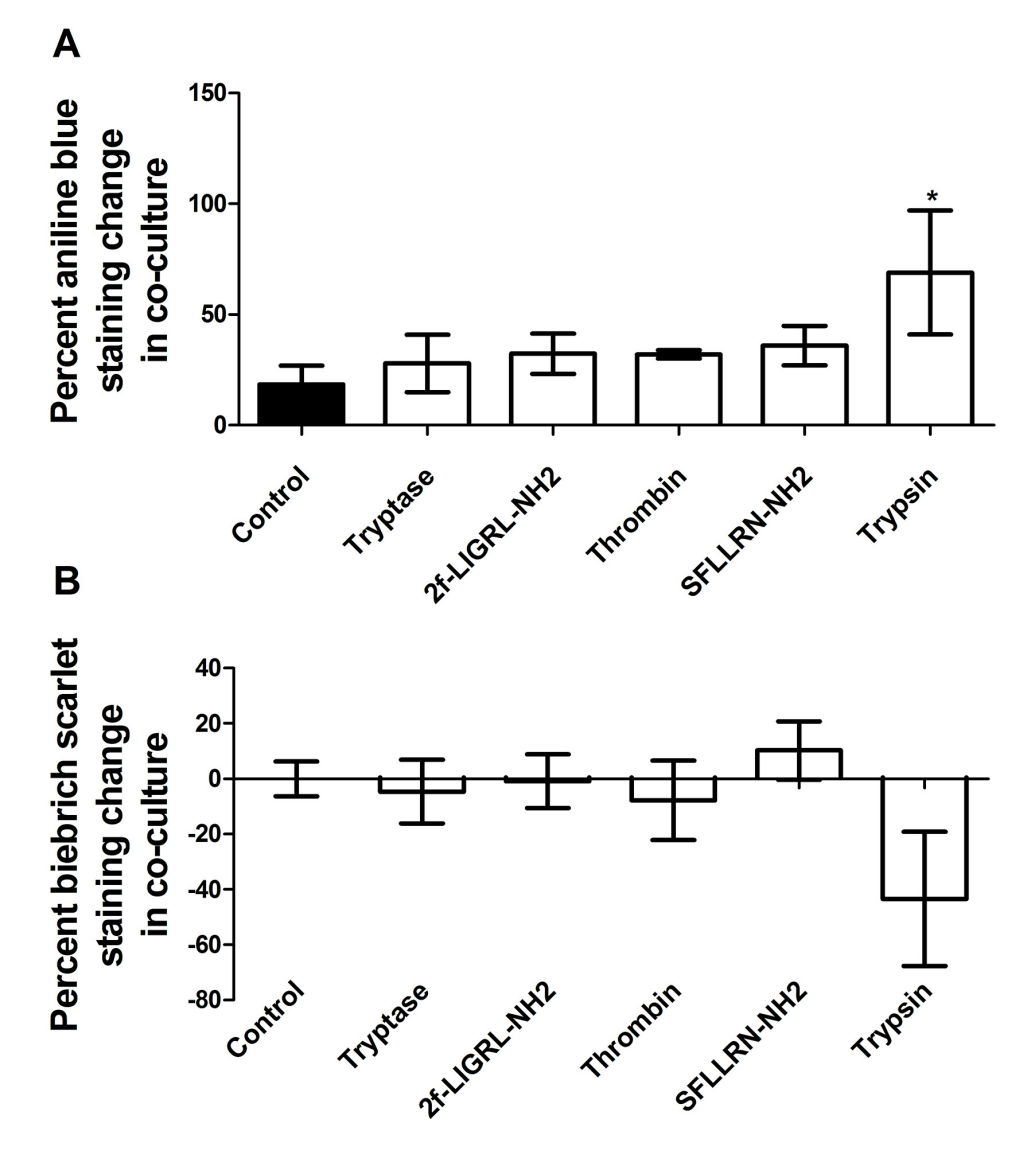
Co-culture of fibroblasts and monocytes increases collagen concentration. PBMC and dermal fibroblasts were co-cultured for five days, either in SFM (control) or in SFM supplemented with a protease or PAR agonist. After five days, plates were stained using Masson’s trichrome, and assayed via absorbance at **(A)** 455 nM for changes in aniline blue staining, which detects collagen, and at **(B)** 700 nM for changes in Biebrich scarlet staining, which stains cells non-specifically. The “percent staining change” was calculated using the equation
$$percent\;staining\;change=\frac{\left(coculture\;absorbance-fibroblast\;absorbance-PBMC\;absorbance\right)}{coculture\;absorbance}\;*\;100$$
Values are mean ± SEM, n = 13 for control, n = 7 for trypsin, and n = 3 for thrombin, tryptase, SFLLRN-NH2, 2f-LIGRL-NH2. * indicates p < .05 compared to the no-protease control (two-tailed t-test).

Collagen is the primary protein component of scar tissue, and is secreted by both fibrocytes and fibroblasts [52]. Proteases increase collagen production by fibrocytes [41] and fibroblasts [9]. To determine if proteases would increase collagen production in a co-culture system, proteases and PAR agonists were added to a co-culture of dermal fibroblasts and PBMC. A modified Masson’s trichrome protocol was used to determine overall collagen secretion. Co-culturing fibroblasts and PBMC produced an 18% ± 8% increase in collagen concentration when compared to the sum of the collagen production of the PBMC culture and the collagen production of fibroblast culture. Each protease and PAR agonist increased collagen concentration above this 18% level, though only with trypsin was this increase significant (Fig 17).

## Discussion

In this report we show that physiological levels of trypsin, tryptase, and thrombin, in addition to acting as pro-fibrotic signals to fibrocytes [18] and fibroblasts [9, 13–15], appear to act as pro-fibrotic signals through PAR1 and PAR2 receptors, altering macrophage surface marker expression and the macrophage secretion profile towards an M2a phenotype. M2a macrophages are involved in scar tissue formation [27–30]. Trypsin, tryptase, and thrombin increased fibronectin staining of macrophages differentiated from monocytes, M1 macrophages, or M2 macrophages. Tryptase and thrombin increased the extracellular IL-4 accumulation in cultures of monocytes, M1 macrophages, and M2 macrophages, while trypsin and tryptase lowered IL-10 and IL-12 accumulation in cultures of M1 and M2 macrophages, respectively. Interestingly, galectin-3 appears to be an excellent marker for M2a macrophages and fibrocytes, and galectin-3 binding protein appears to be a negative marker for fibrocytes, in general agreement with our previous findings [51].

Physiological levels of tryptase and thrombin [18] bias macrophages and monocytes towards an M2a phenotype, suggesting that mast cell degranulation or thrombin activation act to polarize M2a macrophages in both wounds or fibrotic lesions. Physiological levels of tryptase and thrombin also potentiate fibrocyte differentiation [18] and increase fibroblast-mediated collagen deposition [9, 13–15]. Thus tryptase and thrombin are pro-fibrotic signals, and signal through PAR receptors, to each of fibrocytes, fibroblasts, and macrophages, comprising the vast majority of cells in a scar [27–30, 53, 54]. Matrix-metalloproteases, which do not signal through PAR receptors, also influence macrophage polarization [55].

M2a macrophages secrete increased IL-4 and IL-13. IL-4 and IL-13 potentiates fibrocyte differentiation [56] and collagen secretion by fibroblasts [57]. Together, this suggests that trypsin, tryptase, and thrombin are directly pro-fibrotic in their signaling to monocyte, macrophages, and fibroblasts, and are indirectly pro-fibrotic by increasing the amount of IL-4 and IL-13 in wounds and fibrotic lesions. To our knowledge, there is no information to suggest that IL-4 promotes thrombin activation or mast cell degranulation, indicating that trypsin, tryptase, thrombin and IL-4 do not constitute a pro-fibrotic vicious cycle.

Mast cell degranulation lowers the number of M2 macrophages in the local environment without increasing CD163 staining, as we observe [58]. Activated platelets, which are present during the clotting cascade, and thrombin itself increase IL-10 secretion and decrease IL-12 secretion from macrophages [59, 60]. However, fibrin, which also is present during clotting, increases M1 macrophage differentiation, as measured by macrophage inflammatory chemokines [61]. While these findings appear contradictory, there is general agreement that macrophages progress from pro-inflammatory (M1) to anti-inflammatory and remodeling (M2 and M2a) phenotypes during wound healing, so macrophage polarization in a wound depends on an interplay of factors, of which thrombin is only one signal [62–64]. While thrombin is active early in wound healing, mast cells appear more active in the later stages of wound healing [65], and could provide an additional signal to polarize macrophages to an M2a phenotype as the wound is healing. Together, these results indicate that modulating the effects of thrombin and tryptase could be useful to modulate the progression of wound healing and fibrosis.

## Supporting Information

S1 Data (Figures 1-12.pzf). (PZF)

S2 Data (Figures 13-16.pzf). (PZF)

S3 Data (Figure 17.pzf). (PZF)
