# Correction: Trypsin, Tryptase, and Thrombin Polarize Macrophages towards a Pro-Fibrotic M2a Phenotype

**DOI:** 10.1371/journal.pone.0314496

**Published:** 2024-11-20

**Authors:** Michael J. V. White, Richard H. Gomer

In Fig 2, the panel labels are missing and the image of Trypsin under panel B is incorrect. Please see the correct Fig 2 here.

**Fig 2:**
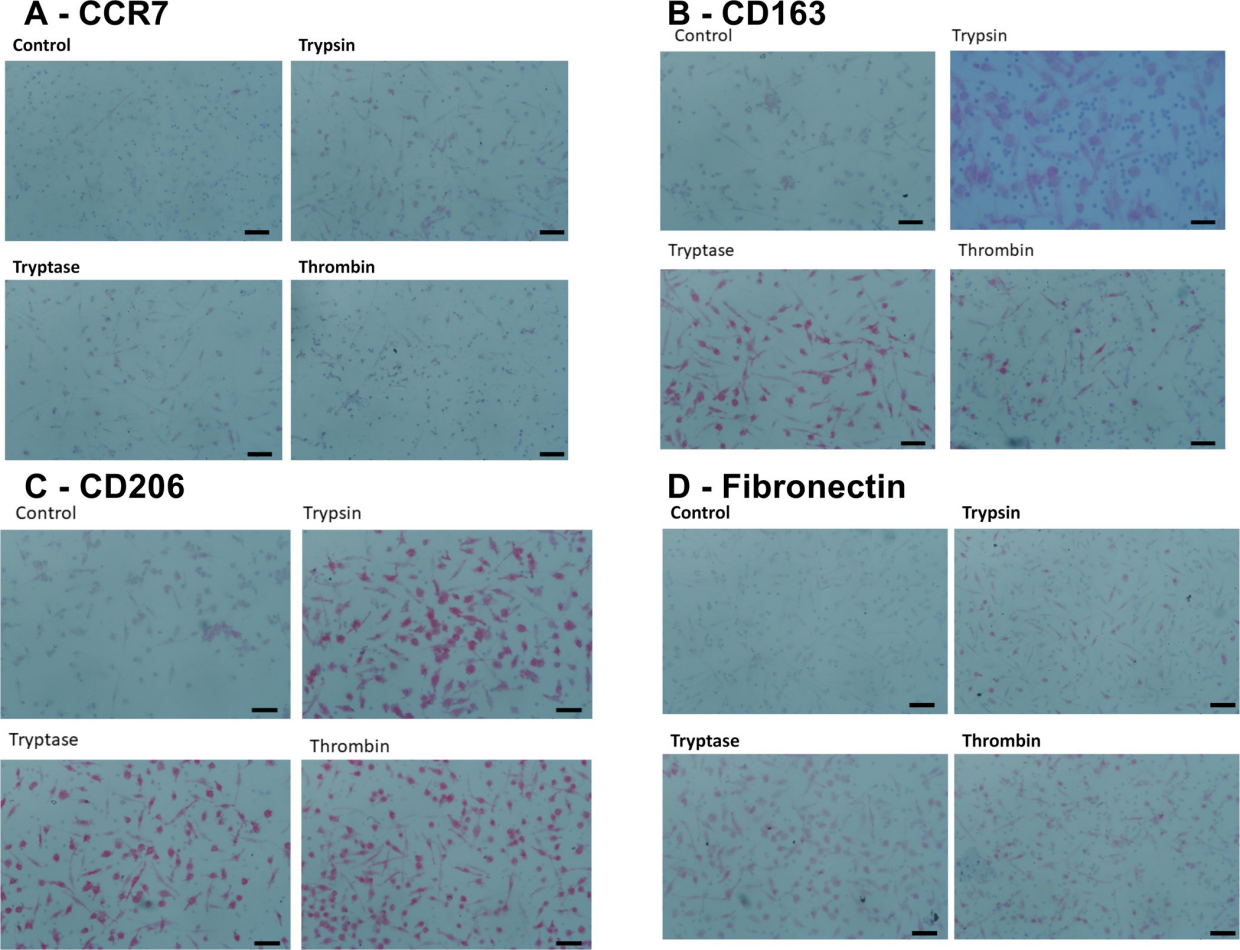
Images of PBMC cultured with proteases. Panels show representative images from slides used for Fig 1, staining for **(A)** CCR7 (mouse monoclonal clone 150503), **(B)** CD163 (mouse monoclonal clone GH1/61), **(C)** CD206 (mouse monoclonal clone 15–2), and **(D)** fibronectin. Bars are 50 μm.

In Fig 6, the image of Trypsin under panel C is incorrect. Please see the correct Fig 6 here.

**Fig 6:**
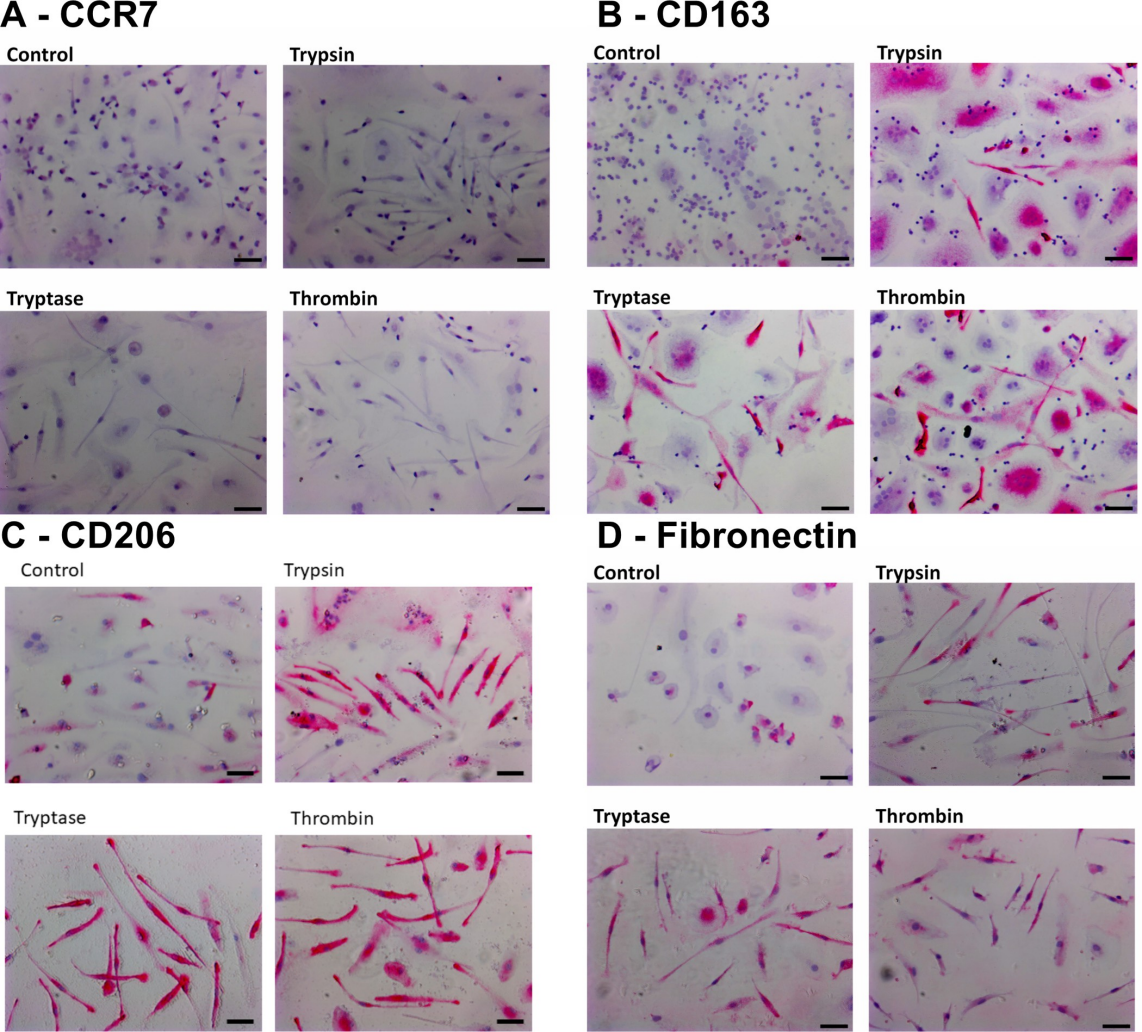
Images of cultures containing M2-biased macrophages subsequently cultured with proteases. Panels show representative images from slides used for Fig 5, staining for **(A)** CCR7 (mouse monoclonal clone 150503), **(B)** CD163 (mouse monoclonal clone GH1/61), **(C)** CD206 (mouse monoclonal clone 15–2), and **(D)** fibronectin. Bars are 50 μm.
